# Time series of heat demand and heat pump efficiency for energy system modeling

**DOI:** 10.1038/s41597-019-0199-y

**Published:** 2019-10-01

**Authors:** Oliver Ruhnau, Lion Hirth, Aaron Praktiknjo

**Affiliations:** 1 Institute for Future Energy Consumer Needs and Behavior (FCN), RWTH Aachen University, Aachen, Germany; 2 Hertie School of Governance, Berlin, Germany; 3 Neon Neue Energieökonomik GmbH (Neon), Berlin, Germany; 4 Mercator Research Institute on Global Commons and Climate Change (MCC), Berlin, Germany

**Keywords:** Energy economics, Energy and behaviour, Energy supply and demand

## Abstract

With electric heat pumps substituting for fossil-fueled alternatives, the temporal variability of their power consumption becomes increasingly important to the electricity system. To easily include this variability in energy system analyses, this paper introduces the “When2Heat” dataset comprising synthetic national time series of both the heat demand and the coefficient of performance (COP) of heat pumps. It covers 16 European countries, includes the years 2008 to 2018, and features an hourly resolution. Demand profiles for space and water heating are computed by combining gas standard load profiles with spatial temperature and wind speed reanalysis data as well as population geodata. COP time series for different heat sources – air, ground, and groundwater – and different heat sinks – floor heating, radiators, and water heating – are calculated based on COP and heating curves using reanalysis temperature data. The dataset, as well as the scripts and input parameters, are publicly available under an open source license on the Open Power System Data platform.

## Abstract (summary)

**Table:** 

Measurement(s)	time sampled measurement data set • heating
Technology Type(s)	computational modeling technique • digital curation
Factor Type(s)	year
Sample Characteristic - Environment	anthropogenic environment
Sample Characteristic - Location	Austria • Belgium • Bulgaria • Czech Republic • Germany • French Republic • Great Britain • Croatia • Hungary • Republic of Ireland • Kingdom of the Netherlands • Poland • Romania • Slovenia • Slovak Republic

Machine-accessible metadata file describing the reported data: 10.6084/m9.figshare.9878849

## Background & Summary

In view of the global energy transition, open energy data are more important than ever^1^. This includes data on electric building heat pumps, which constitute a cornerstone of sustainable energy scenarios^2^. Their power consumption is naturally highly variable. On the one hand, there are fluctuations in the heat demand to be fulfilled by the heat pumps. On the other hand, the COP of the heat pumps, which is defined as the varying ratio of their heat generation and electricity consumption, changes over time. This variability will be essential for the future electricity system balance and needs to be considered in related system and market analyses^3,4^.

Against this background, this paper introduces the When2Heat^5^ dataset comprising the first ready-to-use national time series of both the heat demand and the COP of building heat pumps. The strengths of the dataset include:

1. *Validity*: Historic time series are presented, thus excluding uncertain assumptions on future developments. The heat demand is computed using standard load profiles, which are permanently used by German gas suppliers, and internationally validated with measurements from the UK as well as building data from the EU. The COP calculation is parametrized on manufacturer data and additionally validated with field measurements.
2. *Accuracy*: The dataset considers particularities of different heat demands (space and water heating), different heat sources (air, ground, and groundwater), and different heat sinks (floor heating, radiators, and water heating).
3. *Comprehensiveness*: The time series cover a large geographical area of 16 cold-temperate-climate EU countries (Fig. 1), which is relevant for modelling the balance of the more and more integrated European electricity system. Furthermore, eleven years (2008–2018) are included to enable weather year sensitivity analyses.

**Fig. 1:**
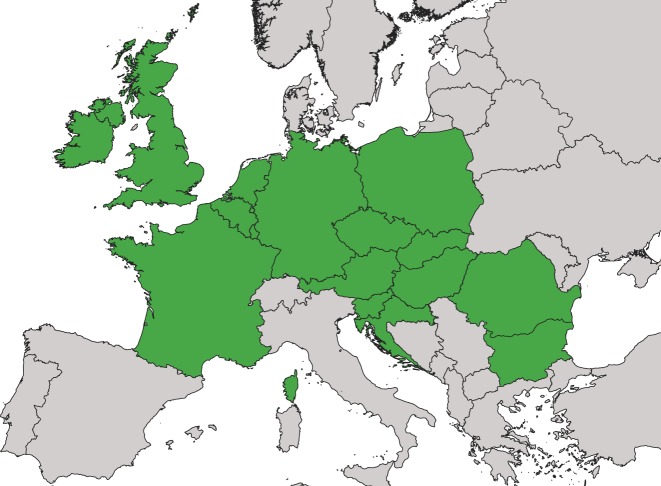
Countries included in the dataset. In alphabetic order: Austria, Belgium, Bulgaria, Czech Republic, Germany, France, Great Britain, Croatia, Hungary, Ireland, Netherlands, Poland, Romania, Slovenia, and Slovakia.
4. *Applicability*: The data are spatially highly aggregated (on the national level), while being temporally highly resolved (hourly). Hence, the format matches those of many European electricity market models.

The *When2Heat* dataset is a contribution to the *Open Power System Data* project and follows the frictionless data principles^6^. Focusing on the representation of heat pumps, the aim is to improve efficiency, transparency, and reproducibility of electricity market models, which might be part of more general integrated energy system analyses. Furthermore, it may serve as a valuable benchmark for alternative heat demand and heat pump modelling approaches on the national level. Existing limitations of the dataset are critically discussed in the Usage Notes section.

**Fig. 2:**
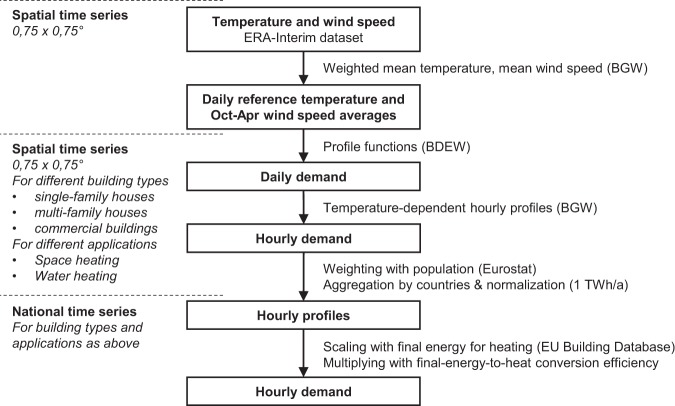
Methodology applied for calculating the heat demand time series. Starting from spatial temperature and wind speed time series from the ECMWF ERA-Interim dataset, gas standard load profiles are derived according to BGW^7^ and BDEW^8^. The time series are weighted with population data from Eurostat GEOSTAT dataset, spatially aggregated, normalized and scaled using the EU Building Database.

The heat demand time series are based on the German gas standard load profile approach defined by BGW^7^ and BDEW^8^. The methodology is combined with three super-national datasets to estimate national time series for 16 European countries. First, temperature and wind speed data from the global ERA-Interim reanalysis serve to generate separate demand time series for space and water heating at all available locations within each country. Next, assuming the heat demand at different locations to be proportional to the population, the spatial time series are weighted with population geodata from Eurostat, aggregated to national time series, and normalized to one TWh average yearly demand. Finally, for the years 2008 to 2013, these are scaled with data on the annual final energy consumption for space and water heating from the EU Building Database and corrected for final-energy-to-heat conversion losses. Figure 2 provides an overview of the methodology applied.

**Fig. 3:**
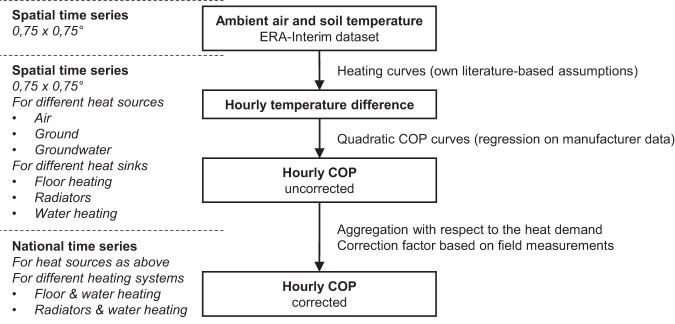
Methodology applied for calculating the COP time series. Spatial temperature time series from the ECMWF ERA-Interim dataset are combined with COP curves based on manufacturer data^9^ to calculate the temperature difference between various heat sources and sinks. After spatial aggregation, the time series are corrected with respect to field measurements^10^.

The COP time series are based on quadratic COP curves, which take the difference between the heat source and heat sink temperatures as an input. The curves are parametrized on manufacturer data^9^ and distinguish between three heat pump types: air-source heat pumps (ASHP), ground-source heat pumps (GSHP), and groundwater-source heat pumps (WSHP). Heat source temperature time series for air and soil are retrieved from the ERA-Interim archive, and a constant groundwater temperature is used. Heat sink temperature time series are computed with literature-based heating curves for floor heating and radiators, and the hot water supply temperature is set constant. Assuming a homogenous adoption of different heat pump technologies across different locations within each country and across various building types, the spatial COP profiles are nationally aggregated with respect to the heat demand. Finally, the time series are corrected based on field measurements^10^. Figure 3 summarizes the methodology applied.

## Methods

This section describes the methodology behind the When2Heat dataset. At first, the data that serve as an input to the calculation of both the heat demand and the COP time series are introduced. Subsequently, the procedures applied for the preparation of the heat demand time series and of the COP time series are presented in detail, respectively. Finally, the code availability is outlined.

### Input data

The time series of the present dataset are based on weather data from the ERA-Interim archive, a global atmospheric reanalysis from the European Centre for Medium-Range Weather Forecasts (ECMWF)^11^. The following parameters are used:

- *2 metre temperature*: The ambient air temperature at 2 m above ground
- *Soil temperature level 4*: The ground temperature at 1.00–2.89 m below ground (https://confluence.ecmwf.int/pages/viewpage.action?pageId=56660259)
- *10 metre wind speed*: The wind speed at 10 m above ground.

The temperature parameters are retrieved for the years 2008 to 2018 in six-hourly temporal resolution, and the wind speed data are retrieved for all available years (1979–2018) in monthly resolution. All parameters feature a 0.75 × 0.75° spatial grid, which is equivalent to approx. 28 × 17 km. For the wind speed, the average of all October-to-April heating periods from 1979 to 2018 is determined for every location, which serves to classify them into “normal” and “windy” locations in the following.

For their spatial aggregation, local time series are weighted with population geodata from the Eurostat GEOSTAT dataset (http://ec.europa.eu/eurostat/web/gisco/geodata/reference-data/population-distribution-demography/geostat). These data originally feature a 1 km² resolution and are thus initially mapped to the 0.75 × 0.75° grid of the ERA-Interim data. For the final scaling of the demand profiles, yearly data on the final energy consumption for space and water heating in residential and non-residential buildings are retrieved from the EU Building Database (http://ec.europa.eu/energy/en/eu-buildings-database).

### Heat demand time series

Temporal heat demand profiles are determined by three factors: weather conditions, building properties, and occupant behavior. Its calculation can follow either statistical methods, including standard and reference load profiles, or physical approaches (for an overview, see Fischer *et al*.^12^). For the When2Heat dataset, the German statistical methodology for calculating gas standard load profiles has been chosen, which is permanently used by gas suppliers for non-daily metered customers. The profiles explicitly refer to space and water heating, and it is assumed that (1) gas boiler operation follows the original heat demand and (2) gas heated buildings are representative for the whole building stock.

The gas standard load profile methodology has been presented by BGW^7^ and updated BDEW^8^. While the calculation of daily reference temperatures is equally included in both references, the calculation of the *daily* demand has been refined in BDEW^8^, and the calculation of wind speed averages (for the assignment of different profiles) and the calculation of *hourly* demand is exclusively described in BGW^7^. Here, both references are used and extended by a spatial dimension to calculate national time series.

Daily reference temperature time series are calculated for each of the ERA-Interim locations. To capture the thermal inertia of buildings, the daily reference temperature, ${T}_{d,l}^{ref}$, is defined as the weighted mean of the daily average ambient air temperature of the actual day, ${T}_{d,l}^{amb}$, and the corresponding temperatures of the three preceding days, ${T}_{d-1,l}^{amb}\ldots {T}_{d-3,l}^{amb}$, for every day, *d*, and every location, *l*^7,8^:

1 $${T}_{d,l}^{ref}=\frac{{T}_{d,l}+0.5\,{T}_{d-1,l}^{amb}+0.25\,{T}_{d-2,l}^{amb}+0.125\,{T}_{d-3,l}^{amb}}{1+0.5+0.25+0.125}$$

**Fig. 4:**
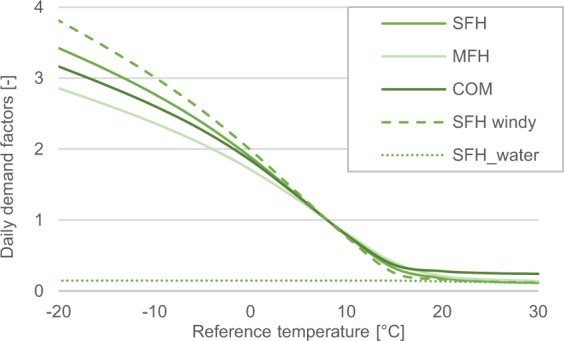
Daily heat demand factors as a function of the reference temperature. Exemplary profile functions for single-family houses (SFH), multi-family houses (MFH), and commercial buildings (COM) and for single-family houses at windy locations (SFH_windy). In addition, daily water heating demand factors for single-family houses are displayed (SFH_water).

Daily demand factors are derived from the reference temperatures using profile functions. These demand factors, *f*_*d,l*_, can be interpreted as unscaled daily demand, which is normalized in the following. The profile function is defined by a combination of a sigmoid function and two linear functions^8^:

2 $${f}_{d,l}=\frac{A}{1+{\left(\frac{B\cdot ^\circ C}{{T}_{d,l}^{ref}-{T}_{0}}\right)}^{C}}+D+max\left\{\begin{array}{c}{m}_{space}\cdot {T}_{d,l}^{ref}{/}^{\circ }C+{b}_{space}\\ {m}_{water}\cdot {T}_{d,l}^{ref}{/}^{\circ }C+{b}_{water}\end{array}\right\},$$

with *T*_0_ = 40 °*C*. BDEW^8^ presents sets of profile function parameters, *A, B, C, D*, *m*_*space*_, *b*_*space*_, *m*_*water*_, *b*_*water*_, for various building types, namely single-family houses, multi-family houses, and commercial buildings. Parameters for more and less temperature-sensitive profiles are provided for different regional weather conditions, which are related to the local wind speed^7^. Therefore, all locations are clustered based on the averaged ERA-Interim wind speed data: for averages above 4.4 m/s, the sigmoid functions for “windy” locations is applied. Otherwise, the locations are assigned to the “normal” category. Figure 4 depicts a selection of the resulting profile functions.

**Fig. 5:**
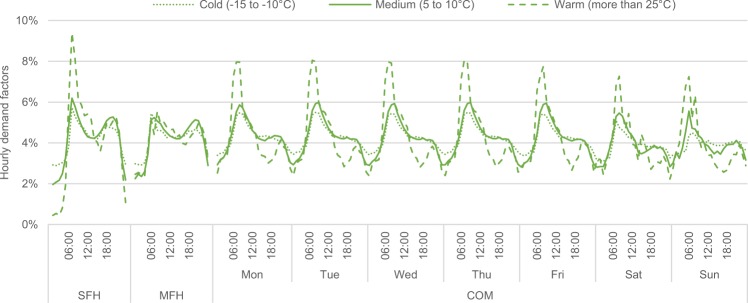
Hourly demand factors at different temperature ranges. Exemplary functions for single-family houses (SFH), multi-family houses (MFH), and commercial buildings (COM). Note that only the factors of commercial buildings depend on the weekday.

Hourly demand time series are derived for each location from the daily values by means of hourly demand factors. BGW^7^ presents these factors for the different building types, ten different temperature ranges, and – in the case of commercial buildings – various weekdays (See page 55 for single- and multi-family houses and pages 85–86 for commercial buildings). Note that different classes are distinguished by the share of old buildings and the type of commerce, but here the German average is considered. These demand factors can be interpreted as hourly shares of the daily demand, i.e. they sum up to 100% per day. For commercial buildings, BGW^7^ additionally derived weekday factors, which scale the daily demand according to the day of the week. Figure 5 depicts a selection of the hourly demand factors, where the weekday factors are already included, i.e. the hourly factors of each day sum up to the weekday factor in the case of commercial buildings.

Separate time series for space and water heating are of interest, e.g., to allow for considering their different temperature levels for the COP calculation. In BDEW^8^, the temperature independent component of the sigmoid function, parameter *D*, and the linear function for water heating, ${m}_{water}\cdot {T}_{d,l}^{ref}+{b}_{water}$, are associated with the gas consumption for water heating. However, the linear water heating function only applies to temperatures at which its value is higher than this of the linear space heating function (see Eq. (2)). This is the case for temperatures above the average heating threshold of approximately 15 °C. For higher temperatures, it is assumed here that the demand for water heating remains constant. Thus, daily water heating demand factors, ${f}_{d,l}^{water}$, are determined at each location according to the following equation:

3 $${f}_{d,l}^{water}=\left\{\begin{array}{ll}D+{m}_{water}\cdot {T}_{d,l}^{ref}{/}^{\circ }C+{b}_{water}, & {T}_{d,l}^{ref} > 1{5}^{\circ }C\\ D+{m}_{water}\cdot 15+{b}_{water}, & {T}_{d,l}^{ref}\le 1{5}^{\circ }C\end{array}\right.$$

Concerning the hourly demand factors, there is no such explicit distinction between space and water heating in BGW^7^. However, assuming there is no space heating at high ambient air temperatures, the hourly demand factors for the highest temperature range (higher than 25 °C) are related to water heating. Hence, daily water heating factors are multiplied with high-temperature hourly demand factors (including weekday factors for commercial buildings) to compute water heating demand time series for each building type. The space heating demand is calculated as the difference between the total heat demand and the water heating demand. Thereby, some negative values occur at the hourly resolution during summer, which are set to zero.

Finally, the resulting spatial demand time series are weighted with the population geodata from Eurostat, aggregated by countries, and normalized to one TWh average yearly demand. Thus, weather year variations cause the exact yearly sum of the normalized time series to fluctuate around one TWh. For the years 2008 to 2013, for which data are available from the EU Building Database, profiles are additionally scaled with the annual final energy consumption for heating. For the residential sector, the demand time series of single- and multi-family houses are aggregated assuming a ratio of 70:30. After scaling, the time series for the residential and non-residential sectors are aggregated for space heating and for water heating, separately. Then, the final energy consumption for heating is transformed to the useful heat demand assuming an average conversion efficiency of 0.9, and the time series are corrected for daylight saving time and different time zones. Space and water heating time series are aggregated in the end, but separate time series are likewise included in the dataset.

### COP time series

The COP of heat pumps generally depends on the temperatures and heat transfer conditions at the heat source and at the heat sink, which are in turn linked to technical properties and varying weather conditions.

**Fig. 6:**
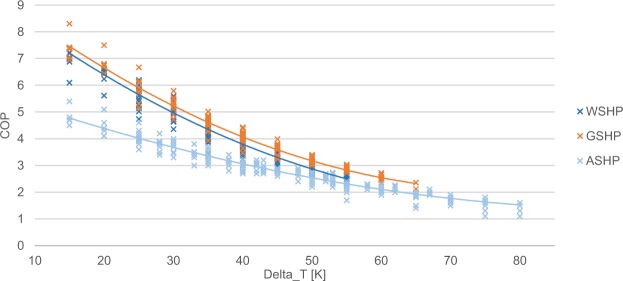
Estimation of COP curves. Quadratic regressions are performed on manufacturer data^9^ distinguishing between air-source heat pumps (ASHP), ground-source heat pumps (GSHP), and groundwater-source heat pumps (WSHP).

The temperature dependence of the COP for the thermodynamically ideal process is described by the Carnot efficiency, which can be scaled down with a quality factor to model real heat pump processes^13^. As an alternative, more generic option for modelling COP variance, quadratic regression has been proposed by Fischer *et al*.^14^. Here, the latter approach is applied to manufacturer data on the COP under different temperature conditions^9^. As shown in Fig. 6, a regression is performed for each of the heat pump types with the following results:

4 $$COP=\left\{\begin{array}{ll}6.08-0.09\cdot \Delta T+0.0005\cdot \Delta {T}^{2}, & ASHP\\ 10.29-0.21\cdot \Delta T+0.0012\cdot \Delta {T}^{2}, & GSHP\\ 9.97-0.20\cdot \Delta T+0.0012\cdot \Delta {T}^{2}, & WSHP\end{array}\right.$$

For simplicity, variable-speed ASHP have not been considered in the regression, i.e. only on-off modulating heat pumps are included. Note that this laboratory-based COP parametrization is adjusted for real-world inefficiencies in the following.

As an input to the COP curves, temperature differences between all possible combinations of heat sources and heat sinks, $\Delta {T}_{h,l}^{sink,source}$, are computed from the heat sink temperatures, ${T}_{h,l}^{sink}$, and the heat source temperatures, ${T}_{h,l}^{source}$, for each hour, *h*, at every location, *l*, using

5 $$\Delta {T}_{h,l}^{sink,source}={T}_{h,l}^{sink}-{T}_{h,l}^{source}.$$

Regarding the source temperature, different heat pump types are distinguished. For ASHP, the ambient air temperature from the ERA-Interim dataset is directly used. For GSHP, the manufacturer data refer to the brine temperature rather than the ground temperature. To account for the heat transfer from the ground to the brine, a temperature difference of 5 K is subtracted from the ERA-Interim ground temperature. For WSHP, a constant temperature of 10 °C and a temperature difference of 5 K for possible intermediate heat exchangers are considered. Hourly values are linearly interpolated between the six-hourly data from ERA-Interim.

**Fig. 7:**
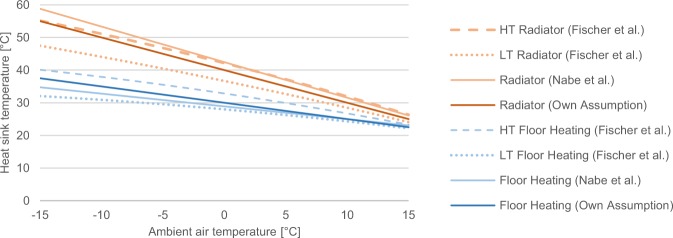
Estimation of heating curves. Own assumptions are compared to literature values form Fischer *et al*.^14^ and Nabe *et al*.^15^, distinguishing between radiators and floor heating systems. HT: high-temperature; LT: low-temperature.

The heat sink temperatures are calculated for floor heating, radiator heating, and water heating. For floor heating and radiators, the temperature time series are derived from the (hourly interpolated) ambient air temperature, ${T}_{h,l}^{amb}$, as described by the following heating curves, which are averages of the existing literature^14,15^, as depicted in Fig. 7:

6 $${T}_{h,l}^{sink}=\left\{\begin{array}{cc}4{0}^{\circ }C-1.0\cdot {T}_{h,l}^{amb}, & radiator\,heating\\ 3{0}^{\circ }C-0.5\cdot {T}_{h,l}^{amb}, & floor\,heating\end{array}\right.$$

In the case of water heating, a constant heat sink temperature of 50 °C is assumed according to German field measurements^10^.

By using these average heating curves, unrealistically small temperature differences are computed at relatively warm outdoor temperatures. To avoid these, a minimum temperature difference of 15 K is introduced, which is in line with the manufacturer data (Fig. 6).

The resulting spatial COP time series, *COP*_*h,l*_, are aggregated to national time series, *COP*_*h,c*_, for every country, *c*, using the following equation:

7 $$CO{P}_{h,c}=\frac{{\dot{Q}}_{h,c}}{{P}_{h,c}}={\dot{Q}}_{h,c}{\left(\sum _{l}\frac{{\dot{Q}}_{h,l}}{CO{P}_{h,l}}\right)}^{-1},$$

where ${\dot{Q}}_{h,l}$ and ${\dot{Q}}_{h,c}$ denote the spatial and national heat demand time series, which are calculated as described above. *P*_*h,c*_ is the national electricity consumption by the heat pumps. For simplicity, the COP time series do not distinguish between different building types, and the sum of the normalized heat demand time series for the different building types is used here. The COP time series for floor and radiator heating systems are spatially aggregated with respect to the space heating demand time series, whereas the COP time series for water heating are spatially aggregated using the water heating demand time series.

A constant correction factor is applied to all COP time series to account for such real-world effects. As shown in the Technical Validation section, the resulting COP time series differ significantly from field measurements. This can be explained by the assumption that the manufacturer data that are used for the COP curve regression are obtained under ideal operating conditions and additional losses will occur in the real world. For example, ideal conditions would imply a steady-state operation at full load, whereas in the real world, the adjustment of heat pump operation to the current demand will be subject to losses. Further inefficiencies may occur from the pumping of groundwater for WSHP and brine for GSHP. The magnitude of the correction factor is set to 0.85, corresponding to field measurements from Günther *et al*.^10^.

## Data Records

All data are available on the Open Power System Data platform^5^. For every country, 24 time series are included in the dataset (Table 1). Nine time series report the absolute heat demand in MW for the different heat applications and building types as well as their aggregation. Note that these time series are available only for the years 2008 to 2013, where data for scaling are available from the EU Building Database. Six profiles cover the normalized heat demand in MW per TWh average yearly demand for the different heat applications and building types. The remaining nine time series represent the COP for different heat pump configurations (three heat sources times three heat sinks). The COP values have no unit or, to emphasize the energy conversion, the unit MW_th_/MW_el_.

**Table 1:** Overview of the 24 time series that are included in the dataset for every country.

Variable	Attribute	Description
heat_demand	total	Heat demand for space and water heating
	space	Heat demand for space heating
	water	Heat demand for water heating
	space_SFH	Heat demand for space heating in single-family houses
	space_MFH	Heat demand for space heating in multi-family houses
	space_COM	Heat demand for space heating in commercial buildings
	water_SFH	Heat demand for water heating in single-family houses
	water_MFH	Heat demand for water heating in multi-family houses
	water_COM	Heat demand for water heating in commercial buildings
heat_profile	space_SFH	Normalized heat demand for space heating in single-family houses
	space_MFH	Normalized heat demand for space heating in multi-family houses
	space_COM	Normalized heat demand for space heating in commercial buildings
	water_SFH	Normalized heat demand for water heating in single-family houses
	water_MFH	Normalized heat demand for water heating in multi-family houses
	water_COM	Normalized heat demand for water heating in commercial buildings
COP	ASHP_floor	COP of air-source heat pumps with floor heating
	ASHP_radiator	COP of air-source heat pumps with radiator heating
	ASHP_water	COP of air-source heat pumps with water heating
	GSHP_floor	COP of ground-source heat pumps with floor heating
	GSHP_radiator	COP of ground-source heat pumps with radiator heating
	GSHP_water	COP of ground-source heat pumps with water heating
	WSHP_floor	COP of groundwater-source heat pumps with floor heating
	WSHP_radiator	COP of groundwater-source heat pumps with radiator heating
	WSHP_water	COP of groundwater-source heat pumps with water heating

The whole dataset is provided in three different file types (csv, xlsx, and sqlite) and three different shapes (singleindex, multiindex, and stacked), following the *Open Power System Data* time series standard. All formats have pros and cons. In contrast to the stacked format, both the singleindex and the multiindex are easy to read for humans and small in file size. The singleindex and stacked files are compatible with the datapackage standard. The multiindex is easy to read into GAMS, which is a widely used technology for modelling electricity markets. All files are indexed by the Coordinated Universal Time (UTC) and Central European (Summer-) Time (CE(S)T) timestamps, indicating the start of the hourly period that the values of each row correspond to.

## Technical Validation

The following two subsections validate the heat demand and COP time series, respectively.

### Heat demand time series

The gas standard load profile methodology applied for the When2Heat dataset is permanently used by German gas suppliers, which confirms its validity for the case of Germany. When transferring the approach to other countries, different local weather conditions are explicitly considered through the spatial ECMWF data. Building properties and occupant behavior, however, are implicitly considered in the parameters of the standard load profile approach, and their possible cross-country variations are not modelled. This section provides evidence that the approach is nevertheless valid for all countries included in the dataset.

The motivation for the creation of the present dataset is the absence of national heat demand time series, which impedes direct validation. However, as already done above, actual data of the non-daily metered gas consumption can be interpreted as a proxy for the temporal profile of the heat demand. Following this interpretation, an exemplary validation is performed for the UK, where such data are available in a daily resolution. Subsequently, the validity of the time series for the other countries included in the dataset is substantiated by induction based on national building insulation statistics.

**Fig. 8:**
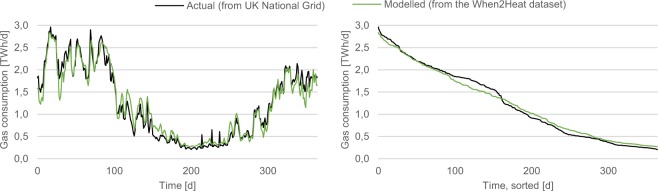
Validation of the heat demand time series for the UK. Modelled UK gas consumption for space and water heating in 2013 are compared to published data from UK National Grid. Daily time series are displayed chronologically (left) and sorted (right).

For the exemplary UK validation of the dataset, actual non-daily metered gas consumption data from UK National Grid (https://mip-prod-web.azurewebsites.net/DataItemExplorer) serve as the benchmark. Daily time series are retrieved for the year 2013, which is the first year fully covered by published data. The yearly sum of the time series amounts to 491 TWh/a, which is assumed to break-down into 365 TWh/a of consumption for space heating, 89 TWh/a of consumption for water heating, and 37 TWh/a of consumption for other purposes, according to DECC^16^. As the present dataset presents heat time series only, the non-heat component is deducted from the National Grid data, assuming a constant temporal distribution. The resulting time series is compared to the sum of the modelled space and water heating demand time series, which are aggregated by days and scaled according to the gas consumption for space and water heating as reported by DECC^16^. Figure 8 compares the temporal profile of gas consumption for space and water heating as reported by National Grid to the modelled temporal profile of the present dataset. Both the chronological time series (left plot) and the load duration curve (right plot) show high consistency (R² = 0.95). This confirms the applicability of the standard load profile approach not only for Germany but also for the UK, even though cross-country variations of building properties and occupant behavior have not been modelled.

**Fig. 9:**
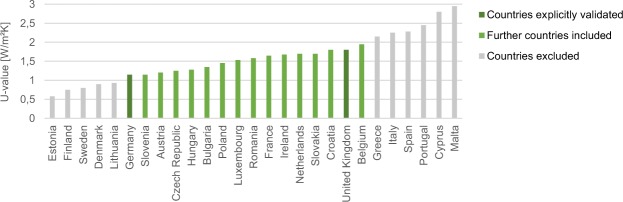
National average heat transfer coefficients (U-values). Different EU countries are compared to assess the transferability of the German gas standard load profile methodology. Countries with values in between or similar to Germany or UK are included in the dataset.

For the other countries included, building characteristics are compared. The most important building characteristic is insulation, which can best be quantified by the heat transfer coefficient. Average national heat transfer coefficients can be retrieved from the EU Building Database (http://ec.europa.eu/energy/en/eu-buildings-database) and are depicted in Fig. 9. Apparently, the insulation of buildings in Germany and UK differs quite substantially but this dataset’s methodology has shown to be robust to this difference and to further differences in building properties and occupant behavior. By induction, it is argued that the methodology will be an adequate estimate for all countries of which the building insulation is in the range of German and UK insulation. This is the case for cold-temperate climate EU countries, which are thus included in the When2Heat dataset. Scandinavian features much better insulation than Germany whereas Mediterranean features much worse insulation compared to the UK, so that these countries are excluded from the dataset.

### COP time series

**Fig. 10:**
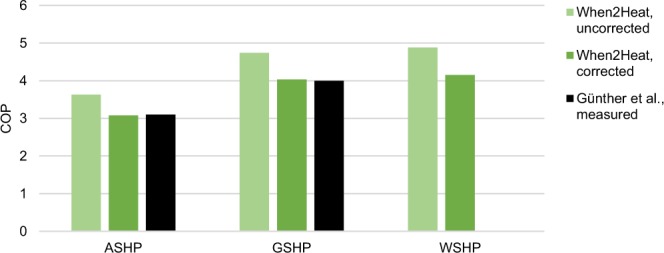
Validation of COP time series. Modelled COP average from July 2012 to June 2013 are compared to field measurements from Günther *et al*.^10^. Air-source heat pumps (ASHP), ground-source heat pumps (GSHP), and water-source heat pumps (WSHP) are distinguished.

To validate the COP time series, the averages are compared to the results of a large-scale monitoring project from July 2012 to June 2013 in Germany^10^. Note that averages are calculated with respect to the demand series and often referred to as performance factors. As the field measurements relate to a portfolio of around 20% water heating and 80% space heating, 85% of which is equipped with floor heating and 15% with radiators, the time series from the When2Heat dataset are aggregated accordingly (analogously to Eq. (7)). As already discussed in the Methods section, the modelled COP is significantly higher than the filed measurements, which is why a correction factor has been applied to increase the validity of the dataset (Fig. 10). For WSHP, Günther *et al*.^10^ have examined only four systems, of which the individual results range from 3.6 to 4.2. The corrected average of the When2Heat dataset is at the top end of this interval, which is satisfactory as the small measurement sample features exceptionally high heat sink temperatures.

## Usage Notes

The following subsections discuss the applicability and the limitations of the dataset, in turn.

### Applicability

The When2Heat dataset is tailored to electricity market models. For a review of power-to-heat modelling approaches, the reader might refer to Bloess *et al*.^17^. Technically, the multiindex format is most convenient for the use in GAMS, which is a widely used technology for modelling electricity markets, but other software tools might be used with the various output formats.

The normalized heat profiles correspond to one TWh average yearly demand. In other words, the exact yearly sum of the normalized time series varies from year to year (around one TWh). In case inter-yearly changes of the yearly heat demand are of interest, the time series should simply be multiplied with assumptions on the average yearly demand. Otherwise, scaling should consider the specific yearly value of the normalized time series.

### Limitations

Critical assumptions were made concerning the spatial aggregation. First, using the population as a proxy for the spatial distribution of the heat demand ignores, e.g., regionally different insulation standards and inhomogeneous per capita living areas. We argue that deriving national time series from population-weighted spatial time series is nevertheless an improvement over using only one representative temperature time series. However, the user might integrate differing weighting assumptions with our publicly available code. Note that an aggregation by other regions, e.g. the European NUTS regions, is also possible through including the corresponding shapefiles. Second, the calculation of the COP profiles assumes homogeneous diffusion within each country and across building types, which neglects the fact that some heat pumps might match certain applications better than others.

Concerning the COP time series, the major simplification is the consideration of average values and constant correction factors. Indeed, the efficiency of individual heat pumps depends on various factors and may deviate significantly from the mean^10,18^. Furthermore, note that the COP is related to the operation strategy of the heat pumps, which might be endogenously determined in the electricity market models. For instance, if heat pumps are optimized with respect to the electricity price by charging a thermal buffer storage during low-price periods, their efficiency will drop due to the higher heat sink temperatures in the buffer storage^19^.

## Data Availability

All code is implemented in Python and published at https://github.com/oruhnau/when2heat under the open MIT license. The entire processing workflow is documented in a single Jupyter Notebook (processing.ipynb), which draws on custom functions that are structured in different Python scripts (download.py, read.py, preprocess.py, demand.py, cop.py, write.py, metadata.py, misc.py). While the download of weather and population data is automated, the input data from the EU Building Database, BGW^7^, and BDEW^8^, as well as the COP curve parameters are included in the code repository.
